# Spatial repellency of transfluthrin-treated hessian strips against laboratory-reared *Anopheles arabiensis *mosquitoes in a semi-field tunnel cage

**DOI:** 10.1186/1756-3305-5-54

**Published:** 2012-03-20

**Authors:** Sheila B Ogoma, Hassan Ngonyani, Emmanuel T Simfukwe, Anthony Mseka, Jason Moore, Gerry F Killeen

**Affiliations:** 1 Ifakara Health Institute, Biomedical and Environmental Thematic Group, Ifakara, PO Box 53, Morogoro, United Republic of Tanzania; 2 Department of Disease Control, London School of Hygiene and Tropical Medicine, Keppel Street, London WC1E 7HT, UK; 3 Vector Group, Liverpool School of Tropical Medicine, Pembroke Place, Liverpool L3 5QA, UK

**Keywords:** Outdoor mosquito control, Spatial repellency, Hessian strips

## Abstract

**Background:**

Vapour phase spatial repellents deter mosquitoes from attacking one or more humans in a protected space. Simulation models indicate that high coverage of spatial repellents can enhance the impact of long - lasting insecticide nets (LLINs) and indoor residual spraying (IRS) where mosquito vectors commonly bite humans outdoors. Here we report a preliminary evaluation of an effective, user-friendly prototype product for delivering spatial repellents to protect against malaria vector mosquitoes.

**Findings:**

Protective efficacy of a 4.0 × 0.3 m strip of hessian sacking treated with 10 ml of transfluthrin was evaluated in a 60 m × 2 m ×2.5 m netting tunnel with malaria-free insectary-reared *Anopheles arabiensis *Patton mosquitoes. Personal protection, in terms of proportional reduction of exposure to bites, was measured by comparing human landing catches of volunteers with treated and untreated strips. A freshly treated hessian strip reduced mosquito attack rate on human volunteers by > 99% and consistently conferred > 90% protective efficacy for a period of 6 months. Over the entire study period, only 22 out of 1400 released mosquitoes bit volunteers using the treated sacking strip while 894 out of 1400 mosquitoes released into cages containing volunteers using an untreated strip fed upon them.

**Conclusion:**

Locally available natural fibers may be promising absorbent substrates for delivering spatial repellents, such as transfluthrin, to protect against mosquitoes in tropical settings. However, these observations relate to a single prototype specimen of this particular device, therefore, much more detailed, well replicated studies are essential to establish long-term efficacy, effectiveness, practicability and affordability.

## Findings

Long lasting insecticidal nets (LLINs) and indoor residual spraying (IRS) have successfully reduced malaria in many endemic regions of Africa [1-4]. These measures have successfully reduced malaria vectors, which predominantly feed upon humans (anthropophagic) and rest (endophilic) and feed (endophagic) indoors [5-11]. Despite impressive successes, these tools are less effective against exophagic, and exophilic mosquito vectors [12,13]. It is therefore critical to find new tools that would protect people whilst outdoors.

Recently developed mathematical models suggest that highly efficacious spatial repellents are likely to be effective when used outdoors in areas where transmission commonly occurs outside of houses [14] or is mediated by mosquitoes which primarily feed upon animals (Kiware et al, Unpublished). Examples of spatial repellent products include mosquito coils and vaporizer mats [15]. Kerosene lamps containing transfluthrin and vegetable oil is a cheap and effective means of dispensing repellents, use of which is well matched to the times and locations of peak human activity [16]. These delivery formats require frequent replacement of the active ingredient and external sources of energy such as combustion or electricity.

Passive methods of delivering spatial repellents without external energy input are highly desirable for impoverished populations in developing countries. Existing products typically consist of paper or plastic strips impregnated with fluorinated pyrethroids, such as metofluthrin or transfluthrin, and have exhibited high efficacy of protection against mosquito bites in some parts of Southeast Asia [17,18]. These pyrethroids are less polar and highly volatile than conventional pyrethroids and therefore evaporate at room temperature without the need for any external source of energy [19]. Such strips can produce vapour for 18 weeks, during which time it repels mosquitoes or prevents them from feeding on humans [18,19]. Interestingly, the level of repellency achieved by treated paper strips has been shown to be more short lived than plastic strips treated in exactly the same manner, demonstrating how different substrates can affect the duration of efficacy exhibited by a given active ingredient [19].

Natural fibers are readily available and affordable in all tropical countries. Initial assessments to compare the physical properties of hessian sacking materials, commonly used for storing and transporting goods in Tanzania, indicated that it had far greater absorbent capacity than commonly available alternatives. The hessian fabric used in this study is made from fine sisal fibers woven together. The fabric is imported from India and is used to make cereal storage bags.

We evaluated the spatial repellency of a hessian sacking strip treated with transfluthrin, in terms of its ability to prevent attack by vectors of malaria in Africa when used outdoors.

Hessian strips 4 m long and 30 cm wide were impregnated with 10 ml technical grade transfluthrin (SC Johnson Home Hygiene Products). A volume of 10 ml of transfluthrin was mixed with 90 ml Axion^® ^liquid detergent (Orbit Chemical Industries Ltd, Nairobi and Colgate-Palmolive East Africa Ltd) to enable its solubility in 400 ml of water. The strips were dipped in the mixture in a plastic basin and suspended indoors at ambient temperature where they were left overnight to dry. A negative control was treated exactly the same way using the mixture of detergent and water only, without any transfluthrin active ingredient.

Experiments were conducted in a screened tunnel measuring 60 m long, 2 m wide and 2.5 m high at the Ifakara Health Institute (IHI) facility in Ifakara, Morogoro, United Republic of Tanzania. The tunnel was divided into three equal-sized experimental units (A, B and C) separated by plastic sheets. Each unit was 20 m long (Moore *et al*. unpublished).

We conducted tests with *Anopheles arabiensis *mosquitoes previously collected from Sakamaganga village, Kilombero valley, South East of the United republic of Tanzania. The mosquitoes were reared in an insectary built within the IHI semi-field system [20]. The temperature in the insectary was between 28 - 29°C and 70-80% relative humidity. Mosquito larvae were fed on tetramin fish food and adults were given 10% glucose solution and blood meals. Nulliparous female, insectary-reared, 2 to 6 day old mosquitoes that had never had a blood meal were used.

Personal protection in terms of the proportion of reduction in mosquitoes attacking volunteers was measured by comparing the number of mosquitoes that landed upon a volunteer with a treated sacking strip and the one who had an untreated strip. Experiments were conducted in units A and C while unit B was used as a buffer zone with no experiments between these two experimental units to minimize the risk that the transfluthrin-treated sacking in one unit would affect mosquitoes in the unit containing the negative control.

**Figure 1:**
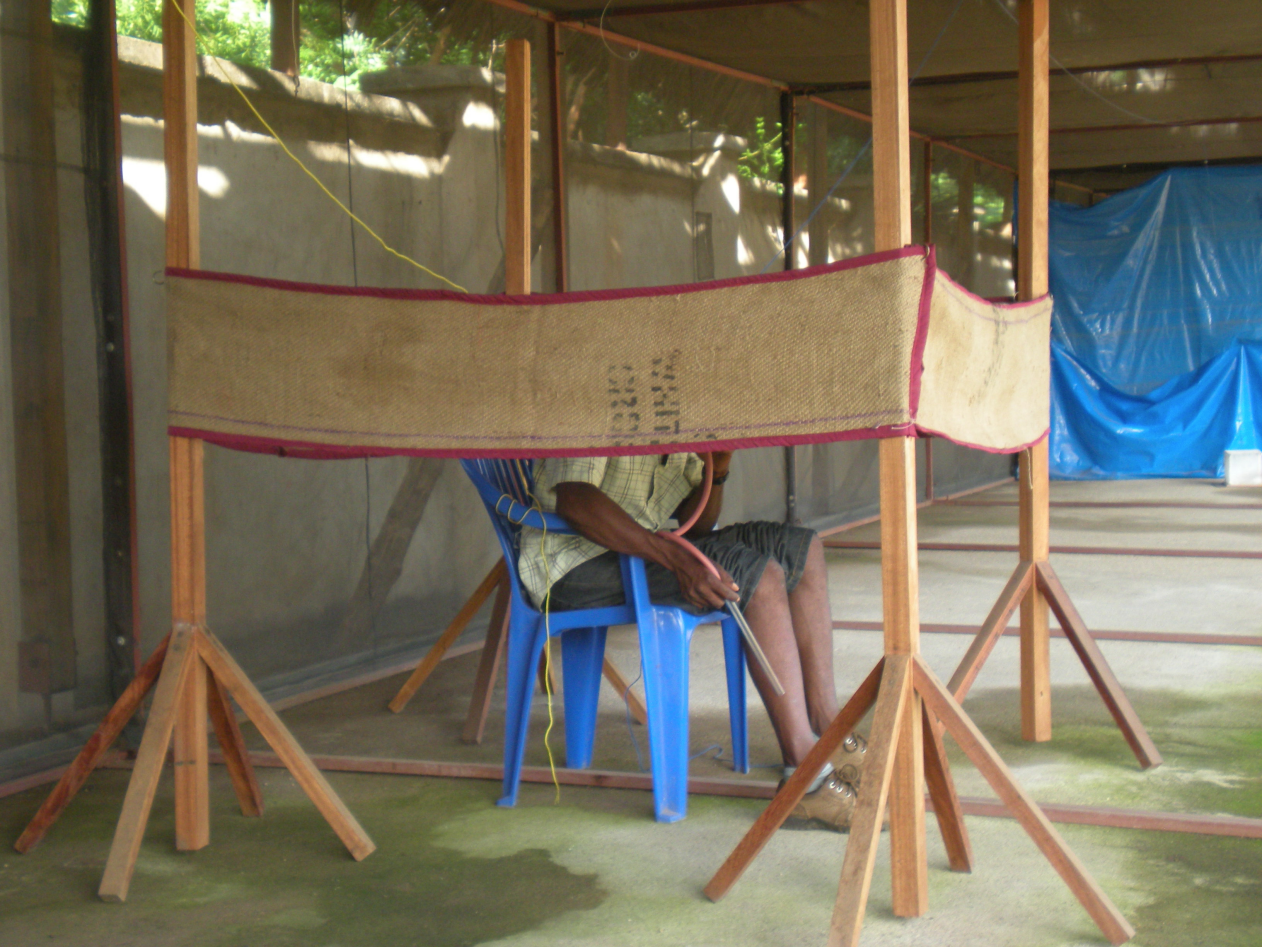
**Transfluthrin hessian strip**. The hessian strip is made from fine sisal fiber woven together to make sacking fabric. The strip is 4 × 0.3 m long. It is treated with transfluthrin. The strip is suspended on 4 wooden poles making approximately 1 m^2 ^area surrounding the human participant conducting mosquito catches.

Each strip was suspended 1 m above the ground in the middle of each unit on a square frame of 4 wooden poles 1 meter apart, thus creating approximately 1 m^2 ^sitting space (Figure 1). Treated and untreated strips were randomly assigned to the units on the first night of every round of 4 nights of experimentation, they were exchanged between units on the third day, and remained in that arrangement for the fourth day. A cage containing 25 mosquitoes was placed at each of the two opposite ends of each unit so that, at the start of the experiment, a total of 50 mosquitoes were released in each unit. Mosquitoes were released at 1900 hours by pulling strings attached to mosquito netting cages placed on each side of the volunteer. Mosquitoes were recaptured by human landing catches simultaneously in both units for 2 hours each night. The two male participants involved in the study were randomly assigned to the experimental units on the first night using the lottery method. They exchanged positions on the second night. On the third night volunteers were randomly assigned to the units again and exchanged positions on the fourth night. Each round of rotation of volunteers and strips between experimental units was completed in 4 nights. One round of experimentation was repeated once every month to check for residual activity of transfluthrin on the hessian strips. The strips were kept in separate plastic basins and stored uncovered at ambient room temperature indoors.

This study was approved by The National Institute of Medical Research (NIMR/HQ/R.8 C/VOL.1/100). Participants signed a written informed consent form before commencing the study.

**Figure 2:**
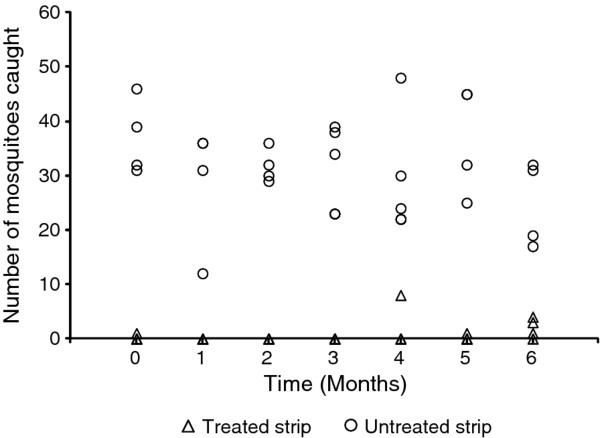
**The number of mosquitoes recovered by human landing catches with transfluthrin and untreated strips**. A graph comparing the number of mosquitoes recovered by human landing catches during rounds of experiments with transfluthrin treated and untreated strips during six months. The graph indicates a reduction in the number of bites occurring on a human participant who had a treated strip compared to one with an untreated strip. Each data point represents a single release of mosquitoes in a single experimental unit with either a treated or untreated strip of hessian sacking.

The freshly treated sisal strip provided > 99% protective efficacy against mosquitoes: In the first round of assays only 1 mosquito out of 200 that were released was recovered by the volunteer in the experimental unit with a treated strip, while 148 out of 200 released mosquitoes were recovered in the unit with an untreated control. The treated strip continued to consistently confer > 99% protective efficacy for a period of 6 months and all assay rounds, except one during the fourth month, indicated approximately 91% protective efficacy. Over the entire study period only 22 out of 1400 mosquitoes released into the experimental unit with the treated sacking strip were recovered by the protected human catcher. In stark contrast, 894 out of 1400 released mosquitoes bit the volunteers using an untreated sacking strip (Figure 2).

While a generalized linear mixed model with a Poisson distribution indicated a clear effect of the treatment status of the hessian strip (P < 0.001), there was no apparent difference between the participants in terms of their attractiveness to mosquitoes (P = 0.208), but the experimental units were significantly different (P = 0.027). The latter effect could be explained by external factors such as light from the nearby security lights shining through one of the units.

Such a prototype conferring such high apparent protective efficacy against outdoor-biting *Anopheles *mosquitoes may well be useful for preventing malaria transmission that mostly occurs outdoors. Our results indicate that hessian sacking substrates may be an efficient means for delivering transfluthrin vapour into an occupied space to protect humans against mosquito bites. Hessian and other natural fibers can be affordably produced in tropical countries, even locally within afflicted communities themselves, thus reducing potential costs of transportation and importation because only the active ingredient needs to be manufactured in bulk by specialist chemical manufacturers. Hessian fibers are a versatile fabric that can be readily woven into a variety of practical formats such as treated wall hangings, door mats or curtains. It might even be possible to weave it into items that can be worn on the body, such as wrist bands, head bands or anklets, so long as the absorbent fiber can be packaged within porous, untreated coating materials that preclude human dermal exposure to the active ingredient.

These preliminary results demonstrated efficacy of transfluthrin strips against mosquitoes under the near-natural conditions of an outdoor semi-field system. However, these observations relate to a single, un-replicated prototype specimen of this particular device [21] so more intensive, well replicated studies in both semi-field systems and full field settings will be required in order to establish these results and characterize the properties of such devices. In particular, it would be important to conduct experiments in which the control and treatment are exposed to mosquitoes alongside each other at a range of proximities within a single semi-field chamber or in full field settings.

The long-term efficacy of the prototype will need to be evaluated at frequent time intervals after formulation and initiation of routine, representative use in target communities. Also, the relationship between protective efficacy and distance from the product will need to be assessed. In particular, the possibility that vapour-phase repellents which prevent mosquitoes from feeding on humans without killing them might pose a risk to nearby non-users by diverting mosquitoes to them [22,23], as is known to occur when using some topical repellents [24] will need to be investigated.

When considering use of spatial repellents, it is necessary to take into account how these can be used with existing tools such as LLINs and IRS in order to complement, rather than reduce, their efficacy [14,22,23,25]. Recently developed models indicate that insecticides which deter mosquitoes from entering houses may undermine the community-level impact upon malaria transmission by the contact toxicity of less volatile conventional pyrethroids applied in the form of LLINs and IRS [14,25]. This is because mosquitoes deterred by sub-lethal doses of an insecticide are prevented from making contact with toxic doses on treated surfaces and are therefore not killed directly. For settings where malaria transmission is dominated, or has historically been dominated, by vectors that typically feed indoors upon humans, it will therefore be essential to assess the mode of action, and community-level impact upon transmission, of products relying upon vapor phase active ingredients when applied both indoors and outdoors to ensure that they complement rather than attenuate the impact of existing front-line LLIN and IRS technologies.

## Competing interests

Two of the authors have received funding and support from the following manufacturers of public health pesticide products: Vestergaard Frandsen SA (GFK) and SC Johnson Home Hygiene Products (SBO). None of these companies played any role in the study design, data collection and analysis, decision to publish, or preparation of the manuscript.

## Authors' contributions

SBO conceived the study, designed experiments, collected and analyzed the data, and drafted the manuscript. GFK supported the formulation of the prototype product, the design of the study, and editing of the manuscript. JM supported the study design and product formulation and constructed the experimental semi-field system. HN, ETM and AM conducted the experiments. All authors read and approved the final version of the manuscript.
